# Chronobiology of epilepsy and sudden unexpected death in epilepsy

**DOI:** 10.3389/fnins.2022.936104

**Published:** 2022-09-07

**Authors:** Benjamin L. Kreitlow, William Li, Gordon F. Buchanan

**Affiliations:** ^1^Medical Scientist Training Program, University of Iowa, Iowa City, IA, United States; ^2^Interdisciplinary Graduate Program in Neuroscience, University of Iowa, Iowa City, IA, United States; ^3^Iowa Neuroscience Institute, University of Iowa, Iowa City, IA, United States; ^4^Department of Neurology, University of Iowa, Iowa City, IA, United States; ^5^Carver College of Medicine, University of Iowa, Iowa City, IA, United States

**Keywords:** epilepsy, circadian, sleep, multidien, SUDEP

## Abstract

Epilepsy is a neurological disease characterized by spontaneous, unprovoked seizures. Various insults render the brain hyperexcitable and susceptible to seizure. Despite there being dozens of preventative anti-seizure medications available, these drugs fail to control seizures in nearly 1 in 3 patients with epilepsy. Over the last century, a large body of evidence has demonstrated that internal and external rhythms can modify seizure phenotypes. Physiologically relevant rhythms with shorter periodic rhythms, such as endogenous circadian rhythms and sleep-state, as well as rhythms with longer periodicity, including multidien rhythms and menses, influence the timing of seizures through poorly understood mechanisms. The purpose of this review is to discuss the findings from both human and animal studies that consider the effect of such biologically relevant rhythms on epilepsy and seizure-associated death. Patients with medically refractory epilepsy are at increased risk of sudden unexpected death in epilepsy (SUDEP). The role that some of these rhythms play in the nocturnal susceptibility to SUDEP will also be discussed. While the involvement of some of these rhythms in epilepsy has been known for over a century, applying the rhythmic nature of such phenomenon to epilepsy management, particularly in mitigating the risk of SUDEP, has been underutilized. As our understanding of the physiological influence on such rhythmic phenomenon improves, and as technology for chronic intracranial epileptiform monitoring becomes more widespread, smaller and less invasive, novel seizure-prediction technologies and time-dependent chronotherapeutic seizure management strategies can be realized.

## Introduction

Epilepsy is a common neurological disease characterized by recurrent and unprovoked seizures (Fisher et al., 2014). Both seizures and epilepsy syndromes are complex, with profound heterogeneity in etiology, presentation, and treatment (Fisher et al., 2017; Scheffer et al., 2017). The mechanisms by which seizures are thought to arise are typically attributed to an imbalance in cerebral excitation and inhibition. When the scales tip toward hyperexcitability, neuronal populations become susceptible to hypersynchronous neuronal activity, which may then manifest as seizures (Scharfman, 2007; Staley, 2015).

Epilepsy is a major public health issue. Approximately one in 26 individuals will develop epilepsy within their lifetime (Hesdorffer et al., 2011a), and current population estimates suggest that over 50 million people live with epilepsy globally (Epilepsy Collaborators, 2019). Unpredictable and difficult to control seizures as well as the effects of anti-seizure drugs can dramatically impair the quality of life for patients who live with epilepsy. Besides accidental injuries that may result from unpredictable seizures, epilepsy is also associated with numerous co-morbid conditions, including mood disorders such as depression and anxiety, sleep impairment, and cardiovascular disease. Persons living with epilepsy are at significantly higher risk of premature death compared to the non-epilepsy population (Levira et al., 2017; Thurman et al., 2017).

There are dozens of anti-epileptic drugs with diverse molecular actions. Unfortunately, these drugs fail to control seizures in nearly one-third of patients with epilepsy (Chen et al., 2018). Patients with drug-refractory epilepsy are at increased risk of sudden death (Devinsky et al., 2016). The phenomenon wherein a person with epilepsy dies following a seizure is known as sudden unexpected death in epilepsy (SUDEP). SUDEP is the sudden and unexpected death of a patient with epilepsy that is not due to trauma, drowning, status epilepticus, or any other concealed pathology that would otherwise be revealed during autopsy (Nashef et al., 2012). The pathophysiological mechanism that leads to death is unclear; however, post-seizure cardiorespiratory demise and impaired arousal are frequently proposed mechanisms (Ryvlin et al., 2013; Devinsky et al., 2016). There is a critical need to better understand the mechanisms that place persons with epilepsy at risk of sudden death.

Although seizures are commonly thought to be unpredictable phenomenon, both historical observations and modern therapeutic technologies suggest this is not the case (Punia, 2021). Our environment changes across days, months, and throughout the year. In response, the body must accommodate these external changes. Biological rhythms that cycle over short timescales, such as the endogenous circadian rhythm and sleep, as well as longer cycles, such as weekly or monthly multidien rhythms, appear to tangibly influence the timing of seizures. How these various biological rhythms influence the timing of seizures is of increasing interest. This is true for SUDEP as well, which has long been considered a primarily nocturnal phenomenon (Buchanan et al., 2021). Historically, these nighttime deaths are typically attributed to seizures that occur during sleep (Nobili et al., 2011; Lamberts et al., 2012), but recent evidence from mouse studies suggest an independent circadian factor is also involved (Purnell et al., 2021b). However, the identity of which biological rhythms contribute to this increased risk of mortality are currently unknown. Clarifying such a circadian contributor could be used to reduce the risk of nocturnal SUDEP, effectively “flattening the curve” of SUDEP risk (Stewart, 2021).

Both seizure timing and the nocturnality of SUDEP are mediated by distinct oscillators that convey risk across different timescales (Baud and Rao, 2018; Amengual-Gual et al., 2019; Rao et al., 2021). Not only will a better understanding of how these rhythmic mechanisms influence seizure timing improve our understanding of *when* seizures occur, they likely will improve our understanding of *why* they occur. Addressing this gap in knowledge sets the stage for new therapeutic strategies, especially as seizure monitoring technology becomes more advanced. Chronotherapeutic seizure management could reduce the incidence of seizures in patients with epilepsy, improving quality of life and overall health and decreasing the risk of premature death.

While there have been several reviews that discuss the influence of biological rhythms on the timing of seizures in the wake of improved long-term intracranial monitoring (Karoly et al., 2021a; Rao et al., 2021), none of these consider how these rhythms may contribute to SUDEP. The purpose of this review is to discuss the current understanding of how different biological rhythms influence the timing of seizures and SUDEP based on both human and animal studies. First, we discuss the timing of seizures with a focus on the multidien and circadian rhythm of onset, with some discussion of menstrual, lunar, and sleep-state contribution. Second, we discuss how two biological rhythms, endogenous circadian rhythms and sleep, may contribute to the nocturnality of SUDEP. Finally, we conclude our review with a brief discussion on the timing of seizure onset and SUDEP and how technological and therapeutic advances that incorporate the biological timing of seizures may be used to improve the quality of life for persons living with epilepsy and decrease the nighttime risk of sudden death.

## Rhythmicity of epilepsy

### Multidien rhythms

**FIGURE 1:**
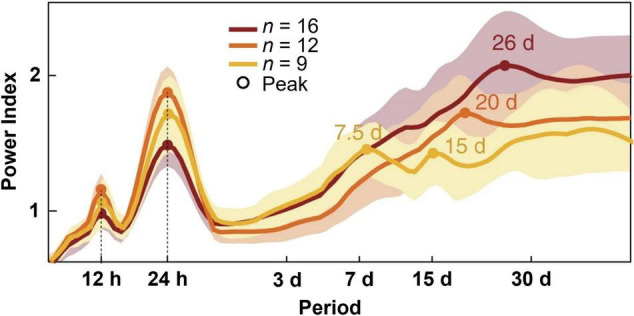
Periodograms of distinct temporal rhythms of interictal epileptiform activity. Recordings from clinically indicated implanted brain stimulators of patients with epilepsy (*n* = 37) reveal ultradian, circadian, and multiple multidien rhythms of interictal epileptiform activity. Shading denotes ± standard deviation. Figure redrawn with permission from Baud et al. (2018).

While circadian rhythmicity is the most studied temporal contributor to seizure timing, rhythms with longer periodicity in seizure susceptibility have also been observed (Figure 1). The periodicity of seizure timing has long been limited by acute recording in clinical environments. With the advent of long-term electroencephalography (EEG) monitoring with implanted, intracranial recording devices, seizure timing over longer timescales can now be readily evaluated (Baud et al., 2021).

Earlier studies from the kainate-induced status epilepticus rat model of epilepsy reveal non-random seizure clustering over longer timescales (weeks/months) (Williams et al., 2009). In a canine model of naturally occurring focal epilepsy, long term intracranial EEG reveals several seizure periodicities, including circadian, weekly, and monthly (Gregg et al., 2020). The timing of these seizures appears to be independent of anti-seizure medication dosing.

Between seizure events, patients with epilepsy often display pathological changes in brain activity known as interictal epileptiform discharges (IEDs) (de Curtis et al., 2012). Findings from patients with implanted brain stimulators capable of detecting IEDs demonstrated ultradian, circadian, and multidien rhythms of these discharges (Baud et al., 2018; Leguia et al., 2021). The observed multidien rhythms could be further sub-categorized into weekly to biweekly, triweekly, and monthly cycles. By studying patients over long timescales in an ambulatory setting, this group was able to show that seizures were most likely to occur during the rising phase of multidien IED rhythms. This technology has since been used to predict seizures days in advanced using previously collected IED data (Proix et al., 2021). Thus, inasmuch as ramping up of these discharges suggests a heightened seizure tendency, rhythms in epileptiform discharge occurrence suggest rhythms in seizure tendency. Although less common, seizures may also fluctuate on circannual (yearly) timescales (Leguia et al., 2021).

The underlying cellular and molecular changes that give rise to multidien seizure risk are poorly understood. Environmental influences, such as changes in photoperiod throughout the year, on individual hormonal and metabolic cycles is one proposed mechanism by which multiple cycles of seizure occur (Rao et al., 2021). Studies in pilocarpine-induced status epilepticus rats suggest endogenous systems, rather than light-dependent changes, mediate the multidien seizure presentation, as animals maintained in identical housing conditions exhibited different multidien cycles (Baud et al., 2019). One proposed endogenous mechanism is the coupling of multidien seizure risk with multidien changes in heart rate (Karoly et al., 2021b). In this study, half of the patients experienced seizures which were coincident with heart rate cycles. However, the observational nature of the study cannot reveal whether this relationship is causal or controlled by a shared mechanism (Karoly et al., 2021b).

### Menstrual cycle

Variability in seizure symptomology around different stages of the menstrual cycle is known as catamenial epilepsy. Alterations in steroid hormones, particularly progesterone and estrogen, at different phases of menstruation are thought to alter cortical excitability (Foldvary-Schaefer and Falcone, 2003).

There is evidence from animal studies that these steroids exert opposing effects (Backstrom, 1976). Animal studies suggest that progesterone and its metabolites are largely anticonvulsant (Holmes and Weber, 1984; Lonsdale and Burnham, 2003). Allopregnanolone, a progesterone metabolite, increases inhibitory GABA_A_ neurotransmission, which may explain its antiseizure properties (Frye, 1995). The effects of estrogens in epilepsy are more complicated. A number of animal studies have shown that estrogens are proconvulsant (Nicoletti et al., 1985; Hom and Buterbaugh, 1986); however, other studies have shown opposing effects (Veliskova et al., 2000; Kalkbrenner and Standley, 2003).

Different concentrations of these steroids at different phases of the menstrual cycle appear to manifest three distinct seizure-clustering periods: perimenstrual (due to decreased levels of progesterone and allopregnanolone), periovulatory (following estrogen surge), and luteal clustering (increased estrogen to progesterone balance) (Herzog, 2015). Adjuvant progesterone therapy reduces seizure frequency in a subset of patients with perimenstrual catamenial epilepsy (Herzog et al., 2012).

### Lunar phase

The mysterious and persistent belief that the phase of the moon may influence human physiology, a phenomenon known as lunar effect, has been of discussion for centuries (Danzl, 1987; Raison et al., 1999). This belief has directed researchers to examine the influence of the phase of moon on seizure timing.

One study observed that patients were more likely to report to the emergency room for seizures during the full moon (Polychronopoulos et al., 2006). Another study similarly observed that lunar phase influenced seizure onset; however, when controlling for clarity of the night sky, based on meteorological data, the relationship was eliminated. In light of this evidence, the authors proposed that these observations were due to nocturnal illumination, and not phase of the moon (Baxendale and Fisher, 2008). A relationship between both the onset of first-time unprovoked seizure or onset of pediatric febrile seizures and phase of the moon has also not been supported (Kim et al., 2019; Wang et al., 2020).

In an attempt to explain this lunar effect on the timing of seizures, some have postulated that changes in nocturnal illumination during different lunar cycles could modulate seizure susceptibility (Baxendale and Fisher, 2008). Increased light during the night could disturb normal sleep or cause sleep deprivation in susceptible individuals, which could trigger seizures. Despite the persistence of this belief, the purported relationship between lunar effect and seizure timing are often inconsistent (Genet and Debes, 2018). As many patients with epilepsy display multidien cycles of seizure timing, some patients are likely to show apparent circalunar seizure timing purely by chance (Rao et al., 2021). The most compelling evidence comes from a group of 222 patients with medically refractory focal epilepsy with implanted responsive neurostimulators (Leguia et al., 2021). While multidien and circadian cycling of seizure timing were highly prevalent, none of the individuals studied had apparent seizure cycling modulated by the phase of the moon.

### Circadian rhythms

The circadian rhythm is an endogenous rhythm that maintains various molecular, physiological, and behavioral processes with a periodicity near 24 h (Moore, 1997). The mammalian central circadian pacemaker resides within the hypothalamus in a region called the suprachiasmatic nucleus (SCN) (Ralph et al., 1990; Hastings et al., 2018).

The SCN maintains daily rhythmicity through a self-regulating transcription-translation feedback loop. Genetic components of the circadian mechanism were initially discovered in *Drosophila* (Konopka and Benzer, 1971; Hardin et al., 1990; Vosshall et al., 1994; Price et al., 1998). The mammalian circadian rhythm is similarly regulated through a set of core clock genes (Panda et al., 2002). Within the nucleus, Circadian Locomotor Output Cycles Kaput (CLOCK) and Brain and Muscle ARNT-Like 1 (BMAL1) transcription factors heterodimerize and promote the transcription of *Per* and *Cry* genes (Gekakis et al., 1998). Period (PER) and Cryptochrome (CRY) then translocate out into the cytosol, slowly accumulate, heterodimerize, and eventually translocate back to the nucleus, where they inhibit CLOCK and BMAL1.

Many different environmental factors are capable of setting the timing of, or entraining, the circadian clock. For example, one powerful time setter, or *zeitgeber*, is environmental light (Yamazaki et al., 2000). Time of day information, in the form of sunlight, is transmitted to the SCN through melanopsin-containing retinal ganglion cells *via* the retinohypothalamic tract (Card, 2000; Hattar et al., 2002). Primary and secondary outputs from the SCN function to synchronize peripheral clocks throughout the body through neuronal and endocrine mechanisms (Yamazaki et al., 2000; Dibner et al., 2010). Destruction of the SCN has been shown to eliminate daily rhythms in corticosterone release (Moore and Eichler, 1972), drinking and locomotive behavior (Stephan and Zucker, 1972), and breathing (Purnell and Buchanan, 2020). While the SCN is conventionally thought to be the master circadian pacemaker, some peripheral clocks may oscillate independently of the SCN and core clock genes. The mechanisms by which these non-canonical circadian rhythms function is of intense interest (O’Neill and Reddy, 2011; O’Neill et al., 2011; Ray et al., 2020).

**FIGURE 2:**
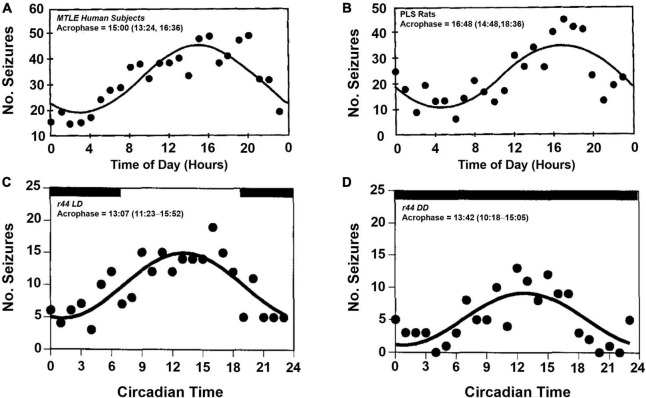
Temporal distribution of spontaneous seizures in humans and postlimbic status epilepticus rats. Spontaneously occurring seizures in a group of human patients with mesial temporal lobe epilepsy (MTLE, **A**) and electrically induced postlimbic status epilepticus (PLS, **B**) rats exhibit time of day dependent timing of seizure onset, with most seizures occurring in the afternoon. Example of a single PLS rat (r44) that experienced frequent seizures throughout the day **(C)**. When housed in constant darkness, the temporal distribution persists, which suggests an underlying, endogenous circadian rhythm regulates time of day dependence of seizure onset **(D)**. Acrophase denotes the time during peak seizure occurrence calculated by cosinor-non-linear least squares (95% confidence limit). Figures redrawn with permission from John Wiley and Sons. **(A,B)** From Quigg et al. (1998). **(C,D)** From Quigg et al. (2000).

Circadian variability in epilepsy has been of interest for nearly a century (Langdon-Down and Russell Brain, 1929; Khan et al., 2018). Early studies of seizure timing had shown that some patients with temporal lobe epilepsy who experienced focal seizures had apparent daily rhythms of seizure onset (Figure 2A), with most seizures occurring during the day (Quigg et al., 1998). In a larger study from the same group, the group’s findings were less clear (Spencer et al., 2016). While most subjects, regardless of seizure foci, had some combination of circadian and ultradian timing of their seizure onset, patients with mesial temporal lobe epilepsy appeared to have equal seizure susceptibility throughout the day. Quigg et al. (1998) also showed that patients with extratemporal lobe and lesional temporal lobe epilepsy, did not have an apparent daily rhythm in seizure onset. The circadian timing of focal seizure onset appeared to be further complicated by the location of epileptic tissue. In a study of patients undergoing intracranial EEG, the distribution of seizures varied throughout the day and were dependent on which region of the brain epileptic foci were found (Pavlova et al., 2004; Durazzo et al., 2008; Spencer et al., 2016). This differential patterning in seizure occurrence based on epileptic focus was also shown in patients in ambulatory care settings (Pavlova et al., 2012). The claim that patients with extratemporal lobe epilepsies do not display daily rhythms has been challenged by more recent studies (Karoly et al., 2016, 2017; Leguia et al., 2021).

Early studies that examined the timing of seizures were limited by the technology available and timescale by which human studies were possible (Baud et al., 2021). With the advent of long-term ambulatory intracranial EEG monitoring (Kossoff et al., 2004; Jobst et al., 2017; Nair et al., 2020), studies on the orders of months (and even years) have clarified the multidien and circadian nature of seizure timing.

In a landmark study that identified multidien timing of seizures in patients with neocortical and mesiotemporal epilepsy (Baud et al., 2018), a number of insights into the circadian timing of seizures were also observed. Although there was clear heterogeneity in the circadian timing of peak interictal epileptiform activity (IEA), the circadian timing could be clustered into three primary groups, with IEAs primarily occurring in the late afternoon, early night, and early morning. Both the circadian and multidien timing of seizures strongly correlated to the rising phase of IEA cycles. Lastly, the group revealed that the greatest risk of seizure coincided when circadian and multidien cycles were in-phase.

In patients with medically refractory focal epilepsy of diverse etiology, chronically implanted continuous EEG and seizure diaries were used to better clarify the prevalence of seizure timing in epilepsy patients across various timescales (Leguia et al., 2021). Firstly, seizure cycling across long time scales are exceptionally common. Most participants exhibited circadian (89%) and multidien (60%) seizure timing. Longer circannual cycles were also observed in a smaller number (12%) of patients, which did not show obvious trends for specific times of year. Like Baud et al., 2018, multidien seizure timing was strongly correlated with the rising phase of the IEA cycle, regardless of the multidien frequency. Interestingly, the circadian timing of the sampled group was more heterogenous than their earlier study, with five distinct clustered groups across the 24-h day (morning, mid-afternoon, evening, early night, and late night).

The independent, but synergistic role of circadian and multidien cycles of IEA was further shown in a post-status epilepticus chemoconvulsant rat model of epilepsy (Baud et al., 2019). Similar to their 2018 study in human patients, peak seizure timing coincides with the rising phase of the IEA cycle in epileptic rats. Careful control of the exposure to light also showed that seizures were most common during the daytime, consistent with previous rodent studies (Quigg et al., 1998, 2000).

Naturally occurring canine models of epilepsy have been used to study the timing of seizures using similar intracranial EEG monitoring used in human patients. Such studies have similarly revealed circadian and multidien seizure periodicities (Gregg et al., 2020). Subsequent studies show that multidien timing of canine seizures preferentially occur during the peak of the IES cycle (Gregg et al., 2021). While several canine subjects had circadian IES cycles, there was significant heterogeneity within the IES rate cycle (i.e., some animals were more likely to experience seizures during the trough, falling, and peak phase of the IES cycle). Within their subjects, animals that experienced multidien cycles of seizure timing were shown to have strong phase overlap, with multidien cycles associated with underlying existing circadian timing.

The induced limbic status epilepticus model of epilepsy in rats show diurnality of seizure susceptibility (Quigg et al., 1998, 2000). In a rat hippocampal kindling model of temporal lobe epilepsy, wherein the brain is rendered epileptic *via* repeated electrical stimulation of the hippocampus, animals exhibit a diurnal pattern of seizure onset, with most seizures occurring during the light-phase (Figures 2B,C). When placing a similar group of animals in constant darkness, the circadian rhythmicity of seizure onset persists with more seizures occurring in the subjective day, i.e., daytime in the absence of light-dark cues, vs. subjective night (Figure 2D).

However, it is not yet clear which genetic or molecular perturbations underlie the circadian rhythmicity of seizure onset. Various studies have shown that disruption of the molecular clock, clock regulating elements, and downstream targets may change the intrinsic properties of neurons or circuits, making them more excitable, and prone to epileptogenic activity.

Quantification of hippocampal mRNA in a rat model of pilocarpine-induced temporal lobe epilepsy has shown that most clock genes, except for *Clock*, vary throughout the day (Santos et al., 2015). Compared to seizure-naïve animals, *Clock* levels were consistently lower in epileptic animals. The reduction of *Clock* in the hippocampus would likely disrupt the local molecular clock mechanism and perhaps render the tissue more susceptible to seizure. A similar study conducted a systematic comparison of seven core clock genes across the 24-h day in a similar seizure model, demonstrating dysregulation in most clock genes (Matos et al., 2018). *Bmal1* continued to oscillate with similar periodicity, albeit with lower amplitude, and *Clock* was apparently arrhythmic.

Utilizing an electroshock seizure induction model, Gerstner et al. (2014) showed that generalized and maximal electroshock seizure thresholds vary throughout the day and are dependent on BMAL1. Interestingly, *Bmal1* knockout animals also have lower seizure thresholds compared to wild-type controls. Patients with temporal lobe epilepsy undergoing surgical resection of epileptic foci have shown decreased levels of BMAL1 in the hippocampal dentate gyrus (Wu et al., 2021). Levels of protocadherin 19 (PCDH19), a cell adhesion molecule regulated by BMAL1, are also decreased in these patients. Familial X-linked mutations in PCDH19 have been shown to result in epilepsy and cognitive impairment in women (Dibbens et al., 2008; Specchio et al., 2011). In patients with hippocampal sclerosis, a common pathology in temporal lobe epilepsy, levels of BMAL1 and PCDH19 are significantly lower compared to patients without sclerosis; however, whether these molecular changes were due to seizure activity or the sclerotic lesion itself are unclear (Wu et al., 2021). Conditional knockout of *Bmal1* in excitatory neurons of the hippocampal dentate gyrus increases seizure susceptibility and mortality (Wu et al., 2021). Altogether, these studies not only show that disruption of clock genes can render one more susceptible to seizures, but also that seizures may disrupt clock-controlled genes and further exacerbate epilepsy symptomology.

Transcriptomic analysis of human epileptic foci has shown decreased levels of *Clock*, similar to rodent studies (Santos et al., 2015; Li et al., 2017). Conditional deletion of *Clock* in excitatory neurons, but not in parvalbumin-expressing inhibitory neurons, reduces the seizure-latency to generalized tonic-clonic seizures following pentylenetetrazol induction in mice. These same mice also experienced spontaneous, sleep-associated seizures, mostly within the first 10 s of sleep onset. The electrophysiological properties of the *Clock* disrupted neurons were also modified, experiencing significantly more epileptiform discharges, which may have been driven by impaired inhibitory input. The morphology of excitatory neurons was also impacted by *Clock* deletion, as affected neurons had reduced spine numbers within apical and 1° dendritic spines, which was similarly observed in epileptic foci from resected human tissue. Despite spine changes, the relative laminar distribution of excitatory cortical neurons was conserved.

The central circadian molecular transcription-translation feedback loop is reinforced by a secondary feedback loop involving REV-ERBα, REV-ERBβ, and RORα, which serves to regulate the transcription of *Bmal1* (Guillaumond et al., 2005). Like CLOCK and BMAL1, expression of REV-ERBα is similarly dysregulated in patients with temporal lobe epilepsy. In both the hippocampus and temporal cortex, REV-ERBα expression is increased epileptic tissue (Zhang et al., 2021). Knocking out REV-ERBα reduced seizure severity and duration in a kainic acid epilepsy model and dampened the kindling process in a hippocampal kindling model. Pharmacologic inhibition of REV-ERBα was also shown to recapitulate the anti-seizure effects from the knock-out mouse model.

The expression of other oscillating genes in the hippocampus are also disrupted in the pilocarpine model, including RORα and melatonin receptors 1 and 2 (Rocha et al., 2016, 2017). Interestingly, the levels of these genes varied throughout the progression of epileptogenesis in this model, with variability observed during the acute, silent, and chronic phases. Understanding how seizures influence temporal expression of RORα, melatonin receptors, and other oscillating genes could serve as adjuvant therapies to address neuroinflammatory changes and melatonergic dysregulation in patients with epilepsy (Bazil et al., 2000b; Vezzani et al., 2019). Members of the PAR bZip transcription factor family show circadian oscillations in the SCN and peripheral tissues (LopezMolina et al., 1997). Deletion of these transcription factors results in spontaneous seizures, with most manifesting shortly after the light-dark transition (Gachon et al., 2004).

Based on previous studies demonstrating time-of-day dependent changes in clock gene expression in the hippocampus, Debski et al. (2020) sought to understand how the downstream targets of cycling clock genes also change throughout the day. Ultimately, they discovered that the transcriptomic and proteomic landscape of the hippocampus changes throughout the day and substantially differed from animals with pentylenetetrazol-induced epilepsy. While wild-type animals had roughly 1,200 oscillating genes, epileptic mice had nearly 1,600, with roughly 500 oscillating genes conserved between the two groups. Genes involved in metabolic control exhibit differential expression between the two groups. Dysregulation of metabolic cycles and its observed circadian nature is thought to be a potential contributor to epilepsy (Chan and Liu, 2021).

### Sleep

Sleep is a period of inactivity characterized by distinct patterns of brain and muscle activity which may be revealed through EEG and electromyography. During sleep, animals transition through different sleep-states, including rapid eye movement (REM) and non-REM (NREM) sleep (Berry et al., 2017). Upon falling asleep, adult humans typically cycle through NREM sleep, which is further sub-divided into stage N1, N2, and N3 sleep based on distinct EEG frequency and amplitude characteristics, and REM sleep throughout the night.

Daily patterning of sleep is understood to be driven by both homeostatic and circadian influences (Borbely and Achermann, 1992; Saper et al., 2005). Studies from human participants housed in a forced desynchrony environment have shown that the periodicity of sleep, and specific sleep states, are under circadian control (Dijk and Czeisler, 1995; Wyatt et al., 1999). Circadian regulation of normal sleep architecture is thought to involve an SCN to ventral subparaventricular zone circuit (Lu et al., 2001).

The role of sleep in epilepsy is of considerable interest (Kellaway, 1985; Mendez and Radtke, 2001; Derry and Duncan, 2013). Dr. William Gower, while working at the National Hospital for the Paralyzed and Epileptic in London, observed consistent sleep-related patterning of seizure onset (Gowers, 1964; Mendez and Radtke, 2001). Gower observed that patients with epilepsy could typically be divided into three categories: those who typically experience seizures during the daytime (42%), randomly throughout the day (37%), or during the nighttime (21%).

There is well-documented evidence demonstrating vigilance state-dependent effects on the timing of different seizure types (Mendez and Radtke, 2001). Several generalized epilepsies often occur upon awakening, most notably juvenile myoclonic epilepsy and generalized tonic-clonic seizures upon awakening, with a subset of generalized tonic-clonic seizures occurring exclusively during sleep, typically in light, NREM sleep (Down and Brain, 1929; Janz, 1962; Bernasconi et al., 1998). Patients with more frequent generalized tonic-clonic seizures are at increased risk of sudden death (Langan et al., 2005; Hesdorffer et al., 2011b).

It is well recognized that focal and generalized IEDs increase during NREM sleep, whereas REM sleep suppresses generalized epileptiform discharges and has a variable effect on focal discharges (Malow et al., 1999; Herman et al., 2001; Rocamora et al., 2013). In addition to increased IED, Minecan et al. (2002) found that NREM sleep is associated with increased seizure frequency, especially during N1 and N2 stages of sleep, even when controlling for the proportion of time spent in distinct sleep states. Their findings mirrored the observations from IED studies, with seizures tending to be more common in NREM than REM sleep. The group also recognized differences in IEDs and seizure occurrence based on the depth of sleep.

While IEDs were more likely to occur during deeper sleep, seizures were more prominent during lighter sleep (N1 and N2). Another group recapitulated Minecan et al.’s findings in a patient population with mesial temporal lobe epilepsy that persisted following anterior temporal lobectomy (St Louis et al., 2004). In the seizure-persistent population, 32% of seizures arose during sleep, with 99% of sleep-related seizures occurring during N1 and N2 sleep (roughly equal proportions). Only a single seizure was observed in slow-wave sleep, and none were observed during REM sleep.

Disorders of sleep are common co-morbidities in patients with epilepsy. Although drowsiness in patients with epilepsy has been attributed to side effects of anti-seizure drugs, disruption of normal sleep also plays a role (Malow et al., 1997; Bazil, 2003). Obstructive sleep apnea significantly worsens seizures in adults (Chihorek et al., 2007), and is often undiagnosed in patients with epilepsy, especially in older men and those who experience nocturnal seizures (Malow et al., 2000). Management of sleep apnea in patients with epilepsy using continuous positive airway pressure reduces seizure frequency in some patients (Malow et al., 2003).

The hypersynchrony of the thalamocortical pathways in sleep has been proposed as a mechanism that facilitates the initiation and propagation of focal seizures (Moore et al., 2021). This hypersynchrony appears to be related to depolarized to hyperpolarized slow wave state transitions during NREM sleep (Frauscher et al., 2015). The microarchitecture of NREM sleep is punctuated by periods of arousal instability (Terzano et al., 2002). This phenomenon, called cyclic alternating pattern, is associated with increased seizure frequency in isolation and in seizure clusters (Manni et al., 2005). In contrast, phasic REM sleep, characterized by physiologic ripples, appears to regularly desynchronize the brain, reducing the number of IEDs during sleep (Frauscher et al., 2016). In patients with temporal lobe epilepsy, seizures, particularly those occurring during the night, were associated with reduced quantity of REM sleep (Bazil et al., 2000a).

## Nocturnality of sudden unexpected death in epilepsy

SUDEP is the leading cause of death in patients with refractory epilepsy (Lhatoo and Sander, 2005; Tomson et al., 2005). It is defined as the sudden and unexpected death of a patient with epilepsy that is not due to trauma, drowning, status epilepticus, or any other concealed pathology that would otherwise be revealed during autopsy (Nashef et al., 2012). Although the pathophysiological mechanisms that proceed death are poorly understood, it is believed that death is heralded by dysfunction in normal respiratory, cardiac, or arousal mechanisms (Kloster and Engelskjon, 1999; Massey et al., 2014; Devinsky et al., 2016).

**FIGURE 3:**
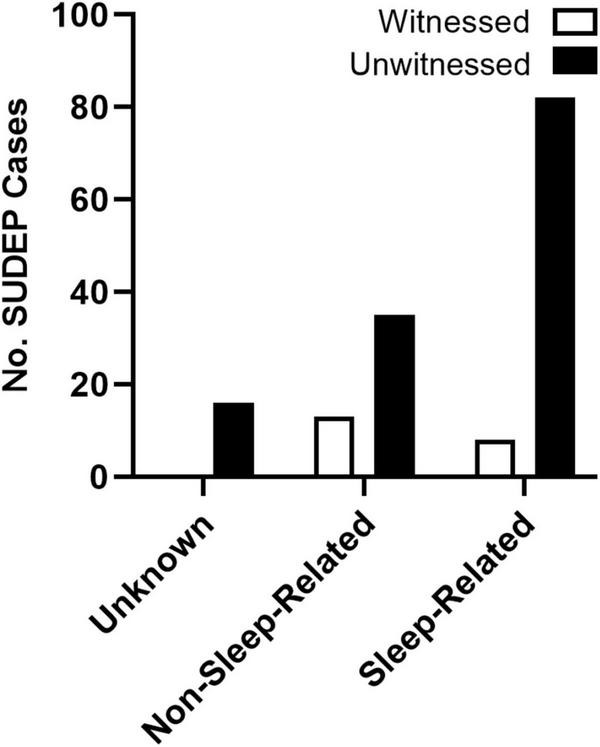
Presumed state of wakefulness during SUDEP events. SUDEP events are often unwitnessed and are frequently associated with sleep. Unwitnessed sleep-related and non-sleep-related seizures were based on the circumstances surrounding death. Events were considered sleep-related if the victim was found in their bed or near their bed with evidence of a recent seizure. For events when victims were not found in the bedroom, SUDEP events were considered non-sleep-related. Events without location or other circumstances surrounding death were considered unknown. Redrawn with permission from John Wiley and Sons from Lamberts et al. (2012).

Despite the controversy surrounding the mechanism of death, SUDEP has long been known to be a largely nocturnal phenomenon. Retrospective studies of the circumstances surrounding death in SUDEP victims consistently demonstrate a nighttime tendency of death (Lamberts et al., 2012; Ryvlin et al., 2013; Ali et al., 2017). Patients who experience nocturnal seizures have also been shown to be at increased risk (Lamberts et al., 2012; Harden et al., 2017). Because victims are often found in or near their beds, this nighttime tendency is frequently attributed to seizures occurring during sleep (Zhuo et al., 2012). However, because the majority of these events are unwitnessed (Figure 3), these studies are almost never accompanied by specific sleep-state information (Lamberts et al., 2012).

Captured SUDEP events from the Mortality in Epilepsy Monitoring Units Study (MORTEMUS) in epilepsy monitoring units support the nocturnal tendency of death, with 14 of the 16 events occurring during the night (Ryvlin et al., 2013). The role of vigilance state, on the other hand, is less clear. While most SUDEP events with video EEG and cardiorespiratory monitoring occurred during sleep, these events did not correspond with any one sleep-state. The isolated role of sleep-state-dependent effects on seizure-associated mortality has also been challenged by recent evidence from mouse studies, which have shown that nocturnal susceptibility to seizure-induced death also has a circadian component that is independent of sleep (Purnell et al., 2021b).

The MORTEMUS study provides some of the most compelling evidence for pathophysiological mechanisms leading to death in SUDEP (Ryvlin et al., 2013). Following a generalized tonic-clonic seizure, victims develop rapid breathing, transient cardiorespiratory changes (episodes of apnea, bradycardia, and/or asystole), terminal apnea, and then terminal asystole. This sequence of events, where respiratory demise always proceeds cardiac demise, suggest that the primary pathological mechanism leading to SUDEP is respiratory in origin. Below, the potential role of sleep and circadian rhythms in SUDEP are discussed.

### Potential sleep-related mechanisms driving sudden unexpected death in epilepsy risk

As all SUDEP events captured from the MORTEMUS study were initiated by a generalized tonic-clonic seizure, it is important to understand how these seizures may impair normal breathing and lead to respiratory demise. Recent studies have described two distinct per-ictal pathways that lead to respiratory failure, ictal central apnea (ICA), and postconvulsive central apnea (PCCA) (Vilella et al., 2019a). ICA appears to be unique to focal epilepsies, whereas PCCA was observed following both focal and generalized epilepsies, which suggests that different mechanisms of seizure-induced respiratory demise may exist (Vilella et al., 2019a; Petrucci et al., 2020). ICA semiologically originates from cortical seizure discharges, whereas PCCA may reflect brainstem dysfunction after a generalized convulsive seizure.

Subsequent studies have shown that the amygdala, hippocampus, and mesial temporal pole act as the symptomatogenic zone for ICA (Dlouhy et al., 2015; Lacuey et al., 2019; Nobis et al., 2019). Activation of the amygdalar network is correlated with central apnea and oxygen desaturation during seizures (Dlouhy et al., 2015). Stimulation of the amygdala can also reliably induce apnea. These studies appear to show that ICA induced respiratory failure is due to the loss of involuntary ventilatory drive rather than dysfunction in respiratory motor control. Taken together, the risk of SUDEP may be higher during NREM sleep, especially during N2. Hypersynchrony of thalamocortical pathways during NREM sleep appear to increase seizure frequency; and if particular seizures impair breathing and cause ICA, these events may be fatal.

Levels of serotonin in the brain appear to be influenced by vigilance state (Python et al., 2001). Levels of serum serotonin appear to be inversely correlated with ICA and PCCA risk (Murugesan et al., 2019). Disruption of brainstem breathing centers during an epileptic event could also link sleep to an increased risk of SUDEP. Unfortunately, recordings of human brainstem seizures are lacking. Using seizure semiology, Vilella et al. (2019b) found a sixfold increase in PCCA risk when seizures presented with postictal brainstem posturing. Because PCCA is associated with SUDEP and near-SUDEP, seizures that spread to the brainstem during low serotonin states, such as during NREM sleep, could increase the risk of postictal respiratory compromise and lead to SUDEP (Patodia et al., 2018; Vilella et al., 2021).

Seizures suppress serotonergic neurotransmission, serotonin levels are inversely related to seizure duration, and selective serotonin reuptake inhibitors reduce seizure associated hypoxemia (Bateman et al., 2010; Murugesan et al., 2018). Furthermore, serotonin regulates respiratory drive by stimulating respiratory output, and serotonin neurons act as central chemoreceptors of CO_2_ and promote brain plasticity in response to intermittent hypoxia (Richerson and Buchanan, 2011). During sleep, there is a rise in CO_2_ during NREM and REM sleep. Ventilatory volume is likely to be responsible for the reduced respiratory drive as the reduction in tidal volume is seen in all stages of sleep and the highest during N3 sleep (Gothe et al., 1981). Respiratory drive is influenced by serotonergic tone through the regulation of breathing and arousal (Saper et al., 2001). Serotonergic raphe activity is highest during wakefulness, reduced during NREM sleep, and silent during REM sleep (Trulson and Jacobs, 1979). This reduction in serotonin level during NREM and REM sleep sets the stage for possible respiratory compromise in SUDEP.

**FIGURE 4:**
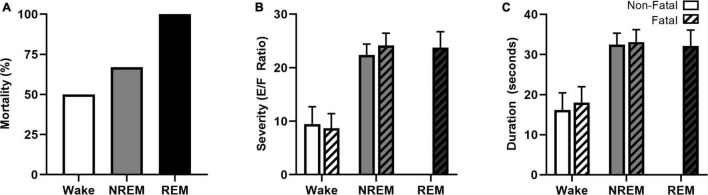
Vigilance state effects on seizure-induced mortality, severity, and duration. Maximal electroshock-induced seizure mortality exhibits vigilance state-dependence, with 50, 67, and 100% mortality for seizures induced during wakefulness, NREM sleep, and REM sleep, respectively **(A)**. Seizures induced during NREM and REM were more severe **(B)** and of longer duration **(C)** than seizures induced during wakefulness. Figures redrawn with permission from Hajek and Buchanan (2016).

The increased prevalence of nighttime SUDEP death and believed deleterious association between nocturnal seizures and sleep have prompted researchers to examine the role that specific sleep-states have on seizure-induced mortality (Hajek and Buchanan, 2016; Purnell et al., 2017). Induced seizure models, including maximal electroshock, allow researchers to control for time-of-day and sleep-state when studying nighttime susceptibility to seizure-induced death. Maximal electroshock seizures are more likely to be fatal when seizures are induced during sleep in the C57BL/6J in-bred mouse strain (Figure 4A; Hajek and Buchanan, 2016; Purnell et al., 2017). Interesting, seizures induced during REM sleep, a state typically believed to be seizure protective (Ng and Pavlova, 2013), were universally fatal. Compared to seizures induced during wakefulness, seizures induced during sleep (NREM and REM) were associated with increased seizure severity (Figure 4B) and duration (Figure 4C) as well as greater respiratory suppression following non-fatal seizures. Despite sleep-state dependent differences in cardiac function at baseline, there were no statistically significant differences in heart rate or heart rate variability between seizures induced during wakefulness and NREM sleep.

### Potential conserved circadian rhythms governing nocturnal sudden unexpected death in epilepsy risk

Although counterintuitive, the time-of-day-dependent activity of the SCN, neurochemical architecture, and molecular machinery maintaining the circadian clock is mostly conserved between nocturnal and diurnal mammals (Smale et al., 2003). The mechanism by which time-of-day specific functions are flipped in nocturnal and diurnal mammals is not well understood. Projections from the SCN to the subparaventricular zone is one proposed mechanism. Disruption of the subparaventricular zone, without damaging the SCN, has been shown to significantly alter the circadian rhythms associated with activity and sleep-state (Lu et al., 2001; Moore and Danchenko, 2002).

Because the circadian rhythmicity of sleep-wake cycles is phase-reversed between nocturnal and diurnal mammals, one could expect seizure-associated death to be similarly phase-reversed. If the nocturnal susceptibility in human SUDEP were exclusively driven by sleep and sleep-state, it would make sense that nocturnal rodents would be more susceptible to seizures that occur during the light-phase of the day, when they are less active and more likely to sleep.

**FIGURE 5:**
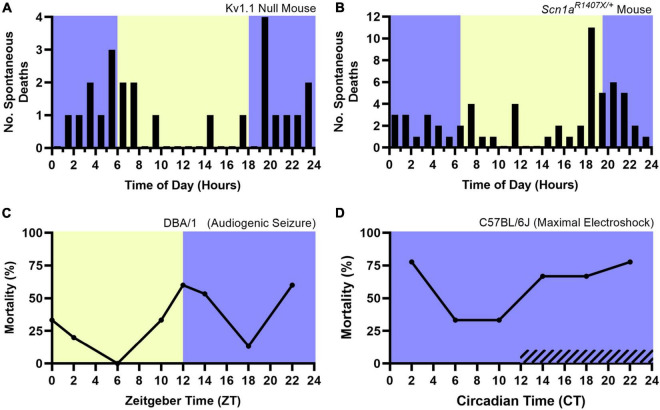
Nocturnal death in spontaneous and induced seizure mouse models. The nocturnal tendency of seizure-associated death has been shown in the Kv1.1 Null Mouse **(A)** and *Scn1a^R140^*^7X/+^ mouse model of Dravet Syndrome **(B)**, both which experience spontaneous, seizure-associated death. Induced seizure models, including audiogenic seizures in DBA/1 mice **(C)** are also more likely to die during the night. When housed in constant darkness, maximal electroshock seizures induced throughout the circadian day in C57BL/6J mice are more likely to be fatal during the subjective night (**D**, hashed lines denote subjective night). Lights on denotes Zeitgeber Time 0. Circadian Time 12 based on onset of locomotive wheel-running activity. **(A)** Redrawn with permission from John Wiley and Sons from Moore et al. (2014). **(B)** Redrawn with permission from Teran et al. (2019). **(C,D)** Redrawn with permission from Purnell et al. (2021b).

Accumulating evidence supports the opposite, as it appears that both models of spontaneous and induced seizure-associated death are more likely to die following seizures that occur during the night. Various chemoconvulsant, electrically induced, and genetic mouse models of epilepsy are being used to study SUDEP (Li and Buchanan, 2019). The Kv1.1 null mouse, a model of temporal lobe epilepsy (Smart et al., 1998), and the *Scn1a^R140^*^7X/+^ mouse model of Dravet syndrome, a severe myoclonic epilepsy with a high rate of SUDEP (Claes et al., 2001; Dravet, 2011), experience spontaneous seizures and seizure associated death. Both mouse models are more likely to die following spontaneous seizures that occur during the night (Figures 5A,B; Moore et al., 2014; Teran et al., 2019). However, these studies lacked vigilance state information, obfuscating the independent role of sleep and circadian rhythms in nighttime SUDEP risk. In addition, it is unclear if potential confounders related to time-of-day, such as reduced environmental activity or noise (i.e., animal caregivers) during the night could contribute to this nocturnal phenotype.

Induced seizure methods allow one to precisely control the time at which seizures occur. The DBA/1 mouse, which is susceptible to audiogenic seizures, and maximal electroshock seizures are two such methods. Purnell et al. (2021b) recently utilized these two seizure induction models to independently examine the role of time-of-day and seizure-associated death. All seizures were induced during wakefulness. DBA/1 mice were housed in a 12:12 light-dark cycle and seizures were induced using an acoustic stimulus at six evenly spaced times of day. Acoustic seizures that were induced during the night were more likely to result in death (Figure 5C). The group conducted a separate experiment, wherein C57BL/6J mice were housed in constant darkness and seizures were induced throughout the circadian day using maximal electroshock. The susceptibility to seizure-induced death exhibited a circadian pattern, with most deaths occurring during the subjective night (Figure 5D). Additionally, it did not appear that seizure severity nor duration or frequency of apneas were influenced by circadian time.

Altogether, these mouse studies reveal a role of time-of-day and circadian rhythms in the nocturnal susceptibility to seizure-associated death that is independent of sleep-state. A number of these seizure models also experience the same pathophysiological cascade observed in SUDEP patients. Similar to SUDEP victims, following a fatal generalized tonic-clonic seizure, mice experience respiratory demise before brain death and eventual cardiac demise (Buchanan et al., 2014; Kim et al., 2018). While the potential circadian mechanism underlying this conserved phenotype is not well understood, a number of potential neurotransmitter systems have been implicated (Purnell et al., 2018).

Diurnal humans and nocturnal rodents share similar oscillations in adenosine (Cornelissen et al., 1985; Chagoya de Sanchez et al., 1993; Chagoya de Sanchez, 1995; Huston et al., 1996), norepinephrine (Linsell et al., 1985; Agren et al., 1986), serotonin (Agren et al., 1986; Rao et al., 1994; Mateos et al., 2009), and melatonin (Ralph et al., 1971; Lewy and Newsome, 1983). The role of adenosine and serotonin are of particular interest in SUDEP pathophysiology (Massey et al., 2014; Petrucci et al., 2020; Purnell et al., 2021a). Both adenosine and serotonin have been shown to have anticonvulsant effects (Boison, 2006; Buchanan et al., 2014) and regulate breathing in response to alterations in blood gases (Koos and Doany, 1991; Hodges et al., 2008; Hodges and Richerson, 2010; Koos, 2011; Iceman et al., 2013). However, adenosine and serotonin appear to have opposite effects on respiratory suppression. Whereas elevated levels of adenosine in the brainstem can impair regular breathing (Barraco et al., 1990), enhancing serotonin neurotransmission can prevent seizure-induced respiratory arrest and death in mouse models of seizure-induced death (Tupal and Faingold, 2006; Faingold et al., 2011).

Convergent evidence from human and mouse studies suggest that the mammalian brain is especially vulnerable to seizures that occur during the night. Sleep-state and circadian rhythms appear to independently influence the nocturnal risk of seizure-associated death. Time of day and circadian physiology are frequently conflated with sleep-state; however, these are biologically distinct phenomena with differential influence on the timing of seizures and seizure-associated death. To better understand the timing of seizures and SUDEP, experiments must be carefully designed so that vigilance state and time of day are independently studied.

## Discussion

Through poorly understood mechanisms, biological rhythms influence the timing of seizures and seizure-associated death. Seizure timing appears to be influenced through endogenous circadian mechanisms, sleep-state-related physiology, and fluctuations in steroid hormones. With the advent of increasingly sophisticated chronic epileptiform monitoring, longer periodic rhythms have been observed in patients with epilepsy.

While suggesting that the time of day, week, or year influences the timing of seizures, or even death, may initially raise eyebrows, the relationship is quite intuitive. Since time immemorial, life has had to adapt to rhythmic changes in the environment over many different timescales, from daily alterations to changes over seasons. These necessary evolutionary adaptations likely carry with them covert effects that influence seizure-susceptibility.

**FIGURE 6:**
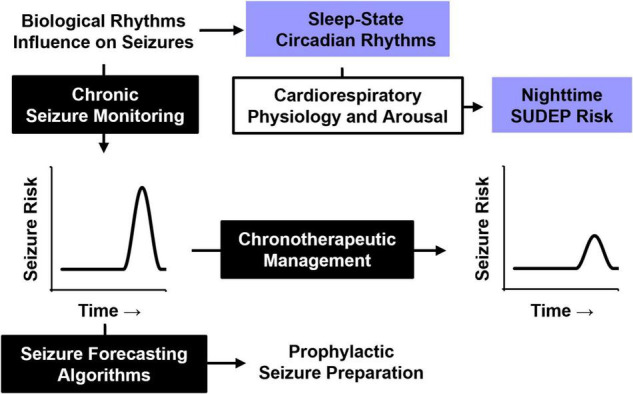
Influence of biological rhythms on seizure timing and SUDEP. A better understanding of how biological rhythms influence seizure timing may be used for future epilepsy management. Periodic seizure risk could be used to forecast future seizures and prepare for them in advance or be used to guide chronotherapeutic medication strategies for at-risk patients. Understanding how nighttime physiology, particularly sleep and circadian rhythms, influence cardiorespiratory and arousal function will likely improve our understanding of SUDEP pathophysiology.

As our understanding of the effect of biological rhythms on seizure timing and SUDEP improve, management of epilepsy will likewise be enhanced (Figure 6). Chronic epileptiform monitoring has been used to predict temporal seizure risk and accurately predict future seizures (Baud et al., 2018; Proix et al., 2021). Understanding the temporal risk of seizure onset in patients also sets the stage for chronotherapeutic epilepsy management.

SUDEP has long been associated with nighttime risk, which is typically attributed to seizures that occur during sleep. However, recent evidence from mouse models suggests an independent role for circadian rhythms, adding a layer of complexity to this nighttime risk. Incorporating the contributions of sleep and circadian rhythms into the management of epilepsy is of increasing interest (Buchanan et al., 2021; Quigg et al., 2021). Understanding how these biological rhythms influence underlying cardiorespiratory and arousal physiology may improve our understanding of SUDEP pathophysiology, and lead to novel strategies to reduce seizure-associated mortality.

## Author contributions

BK and WL drafted the initial document. BK, WL, and GB edited and approved the final manuscript. All authors contributed to the article and approved the submitted version.

## Conflict of Interest

The authors declare that the research was conducted in the absence of any commercial or financial relationships that could be construed as a potential conflict of interest.

## Publisher’s Note

All claims expressed in this article are solely those of the authors and do not necessarily represent those of their affiliated organizations, or those of the publisher, the editors and the reviewers. Any product that may be evaluated in this article, or claim that may be made by its manufacturer, is not guaranteed or endorsed by the publisher.
